# NAD^+^ supplementation rejuvenates aged gut adult stem cells

**DOI:** 10.1111/acel.12935

**Published:** 2019-03-27

**Authors:** Masaki Igarashi, Masaomi Miura, Eric Williams, Frank Jaksch, Takashi Kadowaki, Toshimasa Yamauchi, Leonard Guarente

**Affiliations:** ^1^ Department of Diabetes & Metabolic Diseases, Graduate School of Medicine The University of Tokyo Tokyo Japan; ^2^ Department of Biology, Glenn Labs for the Science of Aging, and Koch Institute MIT Cambridge Massachusetts; ^3^ Chromadex Irvine California

**Keywords:** aging, intestinal stem cells, NAD^+^, nicotinamide riboside

## Abstract

The tissue decline due to aging is associated with the deterioration of adult stem cell function. Here we show the number and proliferative activity of intestinal stem cells (ISCs) but not Paneth cells decline during aging, as does ISC function assessed ex vivo. Levels of SIRT1 and activity of mTORC1 also decline with aging. The treatment with the NAD(+) precursor nicotinamide riboside (NR) rejuvenates ISCs from aged mice and reverses an impaired ability to repair gut damage. The effect of NR is blocked by the mTORC1 inhibitor rapamycin or the SIRT1 inhibitor EX527. These findings demonstrate that small molecules affecting the NAD/SIRT1/mTORC1 axis may guide a translational path for maintenance of the intestine during aging.

## 1 INTRODUCTION

Aging is one of major risk factors of adult‐onset disease such as cancer, diabetes, Alzheimer's and Parkinson's disease, and cardiovascular disease (Niccoli & Partridge, 2012). Adult tissue homeostasis is controlled by adult stem cells, which are continuously proliferative and maintain the tissue (hematopoietic and intestinal stem cells [ISCs]) or quiescent and induced by tissue injury (muscle, liver, and neural stem cells) (Chandel, Jasper, Ho, & Passegué, 2016). Previous studies have described the aging‐related deterioration of adult stem cell function. In certain cases, this decline could be attributed to a loss in the activity of one of the sirtuins, which are nicotinamide adenine dinucleotide (NAD^+^)‐dependent deacylases that regulate aging and age‐related diseases (Guarente, 2013). For example, in hematopoietic stem cells, a reduction in SIRT3 or SIRT7 activity compromises the regenerative capacity of HSCs in aging mice (Brown et al., 2013; Mohrin et al., 2015). In muscle stem cells (MuSCs), reduced amounts of NAD^+^ and the associated decline in activity of SIRT1 are determinants of aging‐related decline. Notably, treatment with NAD^+^ precursor nicotinamide riboside (NR) (Cantó et al., 2012) induces the rejuvenation of MuSCs in aged mice and extends the lifespan of the animals (Zhang et al., 2016).

The rapid turnover of the intestinal epithelium is sustained by ISCs. Previous studies of aging of ISCs mainly come from studies on the intestinal epithelium of *Drosophila *(Biteau, Hochmuth, & Jasper, 2011). During aging, the number and activity of cells that express stem cells marker in *Drosophila* midgut increase due to an environmental challenge or tissue injury (Biteau, Hochmuth, & Jasper, 2008; Choi, Kim, Yang, Kim, & Yoo, 2008; Hochmuth et al., 2011). In mammals, Lgr5‐expressing cells in the base of the crypt constitute the majority of ISCs under normal conditions (Barker, Tan, & Clevers, 2013; Barker et al., 2007). Recently, it was reported that aging results in a decline in ISCs function in mammals and wnt signaling ameliorated the impaired of function of aged ISCs (Mihaylova et al., 2018; Nalapareddy et al., 2017).

Calorie restriction (CR) has been shown to trigger the release of cyclic ADP ribose from the ISC niche, the Paneth cells, (Yilmaz et al., 2012), to drive a pathway of signaling in LgR5‐expressing ISCs to promote their expansion in cell number. This pathway involves SIRT1 activation due to increases in the NAD^+^ biosynthetic enzyme, nicotinamide phosphoribosyl transferase (Nampt), SIRT1 deacetylation of S6 kinase 1 (S6K1), and consequent phosphorylation of deacetylated S6K1 by mTORC1 (Igarashi & Guarente, 2016). Notably, mTORC1 activation by CR in ISCs is opposite to its observed repression during CR in many differentiated cells, including Paneth cells. As predicted by the model, the mTORC1 inhibitor rapamycin and genetic ablation of SIRT1 suppress the effect of CR on ISC expansion (Igarashi & Guarente, 2016).

Here we show the murine ISC number in vivo and functional activity ex vivo decline due to aging. In striking contrast, the number of Paneth cells in mice and their function in supporting ISCs are not impaired by aging. NAD^+^ supplementation by the NAD^+^ precursor NR can rescue these ISC defects and restore a youthful number and function of ISCs. Thus our findings suggest a translational path of NAD^+^ replenishment for rejuvenating the aged gut.

## 2 RESULTS

### 2.1 The ISC pool decreases due to aging

**Figure 1:**
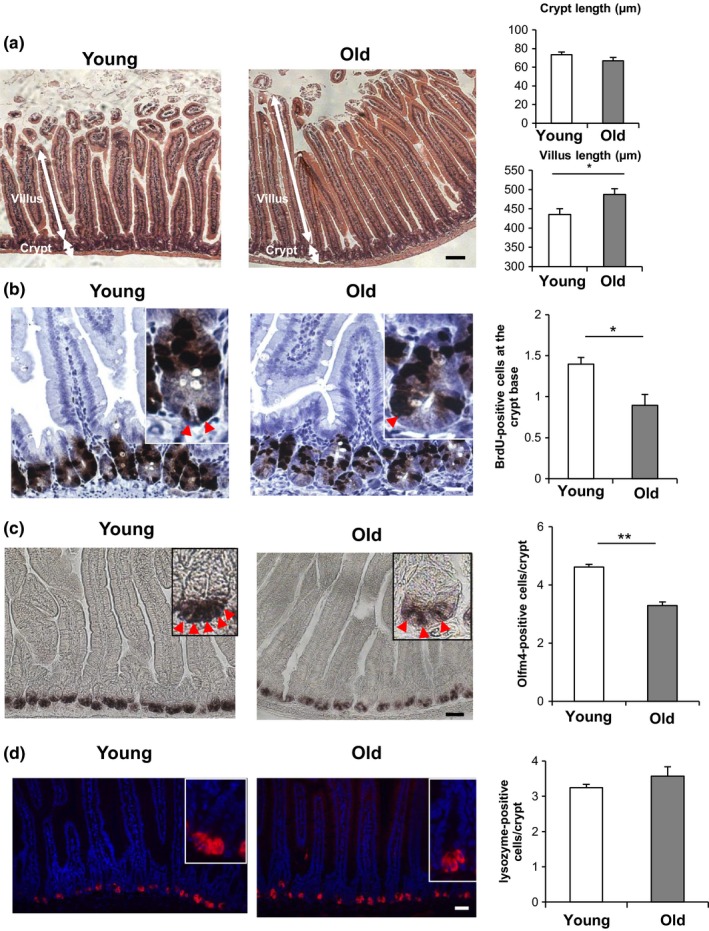
Aging reduces ISC number in vivo. (a) H & E staining images and the quantification of the crypt size and villus size in the intestine of young (3 months old) and old (more than 24 months old) C57BL/6 mice (six mice per group, approximately 50 crypt/villus units per mouse). All histological images in this and subsequent figures are typical of numerous tissue samples analyzed. (b) BrdU staining images and the quantification of BrdU‐positive crypt base columnar (CBC) cells (arrowheads) adjacent to Paneth cells at the bottom of crypts as assessed 2 hr after the injection of BrdU (three mice per group, 50 intact well‐orientated crypts per mouse). (c) In situ hybridization images of Olfm4 mRNA and the quantification of Olfm4+ ISCs (arrowheads) (three mice per group, approximately 25 intact well‐orientated crypts per mouse). (d) Lysozyme staining image (Red: lysozyme, Blue:DAPI) and the quantification of lysozyme‐positive Paneth cells (three mice per group, 50 intact well‐orientated crypts per mouse). Original magnifications: ×100 (a); ×400 (b); and ×200 (c and d). Scale bar: 100 µm (a); 25 µm (b); and 50 µm (c and d). Values represent the mean ± *SEM*. *p < 0.05; **p < 0.01; *t* test. ISC, intestinal stem cell

To assess the effect of aging on gut homeostasis, we compared the histology of small intestine in young (3–5 months old) and old (more than 24 months old) C57BL/6 mice. Morphologically, aging induced an increase in villus length and crypts showed a trend to a smaller size, which did not reach significance (Figure 1a). In agreement with the increase in villus size, aging increased the numbers of differentiated cells of the gut: absorptive enterocytes, goblet cells, and chromogranin A+ enteroendocrine cells (Supporting Information Figure S1a–c).

Next, to test whether aging influences the population of proliferative cells in young versus old mice, 2‐hr BrdU labeling was performed in crypts, which marks ISCs and their immediate descendants, transit amplifying (TA) cells (Figure 1b and Supporting Information Figure S1d). There was no difference between young and old mice in the incorporation of BrdU into total crypt cells (ISCs and TA cells) (Supporting Information Figure S1d). However, there was a decrease BrdU in incorporation into the crypt base columnar (CBC) cells, which are stem cells wedged between Paneth cells (Figure 1b) (Barker, van Oudenaarden, & Clevers, 2012), indicating that proliferation of ISCs decreased in old mice compared to young mice. Next to investigate the number of ISCs in young versus old mice, we performed in situ hybridization (ISH) for Olfactomedin‐4 (Olfm4), which is a marker of ISCs (van der Flier, Haegebarth, Stange, van de Wetering, & Clevers, 2009; Flier et al., 2009). The number of Olfm4+ positive ISCs in the crypts of old mice decreased compared with young mice from about five cells per crypt to about three cells per crypt (Figure 1c). A similar finding was noted using FACS analysis of mice expressing Lgr5‐GFP+cells to count ISCs (Supporting Information Figure S1e). Paneth cells are niche cells which are adjacent to and support the proliferation of ISCs (Sato et al., 2011). Particularly, ISC proliferation in CR depends on the cyclic ADP ribose secreted from Paneth cells (Igarashi & Guarente, 2016; Yilmaz et al., 2012). However, we did not find any difference in the number of Paneth cells (lysozyme positive) between young versus and old mice (Figure 1d). These results all indicate that the number of ISCs in old mice decreases and the villi length increases, suggesting that self‐renewal of ISCs is reduced and differentiation of ISCs is consequently increased in old mice.

### 2.2 Aging degrades the formation of intestinal organoids from ISCs

**Figure 2:**
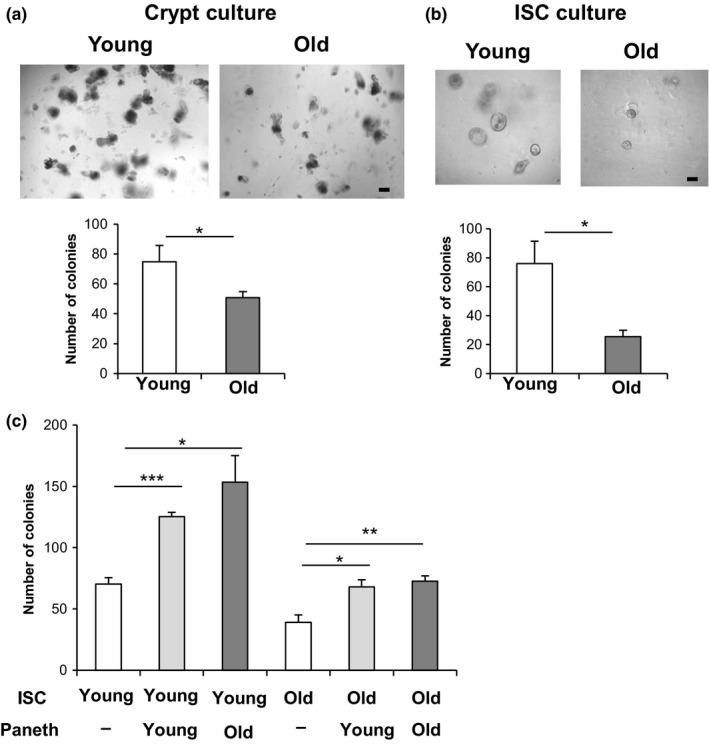
Aging reduces the formation of intestinal organoids from ISCs ex vivo. (a) Crypts from young and old mice were cultured in Matrigel to allow ISCs to form organoid colonies. The number of colonies was assessed at day 5 (7–8 wells/group (the sum of two different experiments). The picture shows colonies cultured from young crypts and old crypts. (b) ISCs were isolated from young and old *Lgr5‐EGFP‐IRES‐CreERT2* mice (>95% pure), and 2 × 10^3^ cells were cultured in the absence of Paneth cells in culture medium containing 10 µM CHIR. The number of colonies was assessed at day 5 (11–15 wells/group [the sum of three different experiments]). The picture shows colonies from young ISCs and old ISCs culture in the absence of Paneth cells. (c) ISCs and Paneth cells were isolated from young and old *Lgr5‐EGFP‐IRES‐CreERT2* mice, and 2 × 10^3^ cells each were co‐cultured in the medium containing 10 µM CHIR. The number of colonies was assessed at day 5 (3–4 wells/group). Original magnifications: ×100 (a and b). Scale bar: 100 µm (a and b). Values represent the mean ± *SEM*. *p < 0.05; **p < 0.01; ***p < 0.001; *t* test. ISC, intestinal stem cell

To further investigate the effects of aging on the proliferation of ISCs, we isolated crypts from young and old mice, dispersed the cells, and seeded them on Matrigel to obtain organoid colonies (Igarashi & Guarente, 2016; Sato & Clevers, 2013). Each organoid colony is seeded by a single ISC and gives rise to all the differentiated cells of the gut in the colony. This assay thus enumerates the functional ISCs ex vivo. Crypts from old mice formed fewer organoid colonies and showed a decreased number of buds，which is another indicator of stem cell function, compared to those from young mice, consistent with the in vivo data (Figure 2a and Supporting Information Figure S4a). Next to assess organoid‐forming ISCs more accurately and address how the ISCs and Paneth cells interact functionally, we isolated Lgr5‐positive ISCs and Paneth cells each to >95% purity from young and old *Lgr5‐EGFP‐IRES‐CreERT2* mice, as described previously (Igarashi & Guarente, 2016). A total of 2,000 cells were plated of each cell type and in the standard media containing glycogen synthase kinase 3β (GSK3β) inhibitor CHIR99021 (CHIR), which is known to induce β‐catenin and thus stimulate organoid formation (Igarashi & Guarente, 2016; Yin et al., 2014). Lgr5‐positive ISCs isolated from old mice formed fewer and smaller colonies on day 5 of culture and fewer organoid colonies with a decreased number of buds on day 9, compared with those from young mice (Figure 2b and Supporting Information Figure S2a and S5b,c). By combining young or old ISCs and young or old Paneth cells, we determined that the Paneth cells from old mice were as functional as young Paneth cells in stimulating organoid colony formation in co‐culture with young ISCs and with or without CHIR (Figures 2c, Supporting Information Figures S2b and S5d). Old ISCs were similarly defective in organoid formation with either young or old Paneth cells. Thus, this functional ex vivo assay for ISC function and niche–stem cell interaction demonstrates a defect in old ISCs but not old Paneth cells.

To gain insights into the mechanisms driving loss of organoid formation in old ISCs, we focused our analysis on gene networks previously associated with ISC stemness (Igarashi & Guarente, 2016) and found a significant reduction in SIRT1 protein in old crypts and ISCs (Supporting Information Figure S2c,d). Also, we observed a trend to a reduction in S6 phosphorylation in old crypts and old ISCs, indicative of a lowering in SIRT1/mTORC1 activity (Figure 4c,d). Notably, RNAseq analysis of young and old ISCs did not reveal significant differences in gene expression, suggesting that relevant changes occur post‐transcriptionally (Supporting Information Figure S3).

### 2.3 Nicotinamide riboside restores the colony formation in aged mice

**Figure 3:**
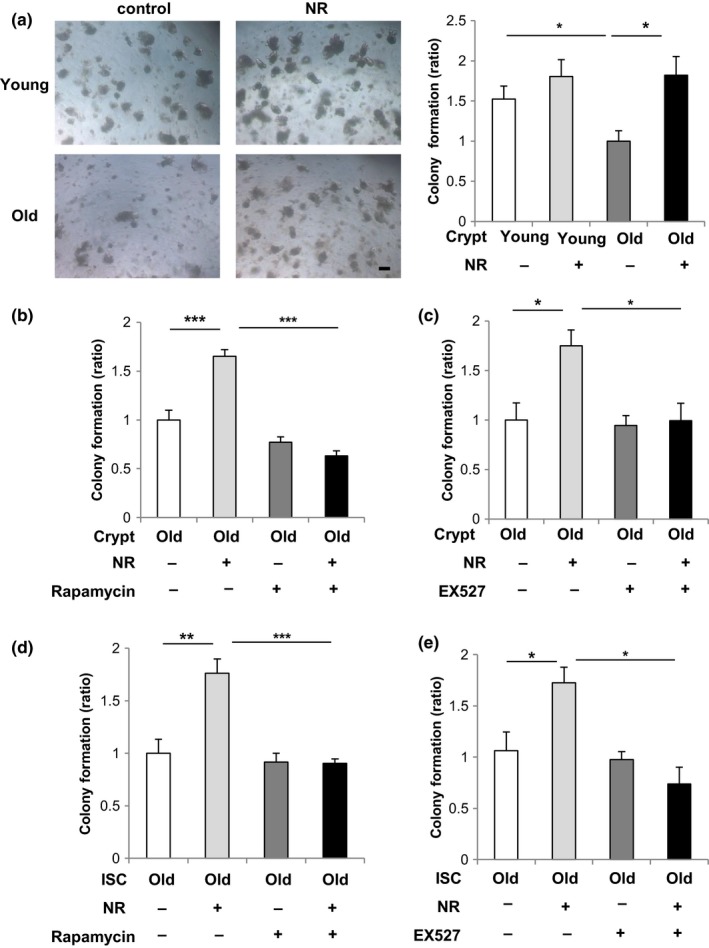
Nicotinamide riboside (NR) restores the colony formation in aged mice. (a) Isolated young or old crypts were cultured in medium with or without 1 mM NR as indicated (5–6 wells/group). Representative images of the formed organoids at day 5 and the quantification of organoids number. Original magnifications: ×50. Scale bar: 100 µm. (b) Isolated old crypts were cultured in medium with or without 1 mM NR and 1 mM rapamycin as indicated (five wells/group). (c) Isolated old crypts were cultured in medium with or without 1 mM NR and 1 µM EX527 as indicated (4–5 wells/group). (d) Isolated old intestinal stem cells (ISCs) were cultured in medium with or without 1 mM NR and 1 mM rapamycin as indicated (four wells/group). (e) Isolated old ISCs were cultured in medium with or without 1 mM NR and 1 µM EX527 as indicated (three wells/group). Values represent the mean ± *SEM*. *p < 0.05; **p < 0.01; ***p < 0.001; *t* test

In order to probe the role of the SIRT1/mTORC1 axis ISC aging, we tested whether NAD^+^ supplementation affects the function of old ISCs ex vivo. Thus, we treated crypts from young and old mice with NR, a NAD^+^ precursor, which increases intracellular NAD^+^ levels and activates SIRT1 (Cantó et al., 2012). NR treatment completely rescued the diminishment in colony formation efficiency and number per organoid of differentiated buds in old crypt‐derived organoids, while having no significant effect in the colonies from young crypts (Figure 3a and Supporting Information Figure S4a). To address in more functional detail the role of the SIRT1/mTORC1 pathway in the NR‐rejuvenated ISCs, old crypts were incubated with NR plus the mTORC1 inhibitor rapamycin or the specific SIRT1 inhibitor EX527. Rapamycin or EX527 treatment of old crypts blocked the rescue of organoid formation by NR treatment (Figure 3b,c), while having a minimal effect on NR‐treated young crypts (Supporting Information Figure S4b,c). Next to be certain these effects occurred specifically in the ISCs, we purified Lgr5+ stem cells and carried out parallel experiments to the studies using crypts. We found with the pure ISCs that NR again increased organoid colony formation by old ISCs, and that this increase was blocked by rapamycin or EX527 treatment (Figure 3d,e), while having a minimal effect on NR‐treated young ISCs (Supporting Information Figure S4d,e). These studies show that ISC functional decline with aging can be rescued by NAD^+^ replenishment via NR and that the effect is intermediated by SIRT1/mTORC1 signaling pathway.

### 2.4 NR treatment restores ISC number in aged mice in vivo

**Figure 4:**
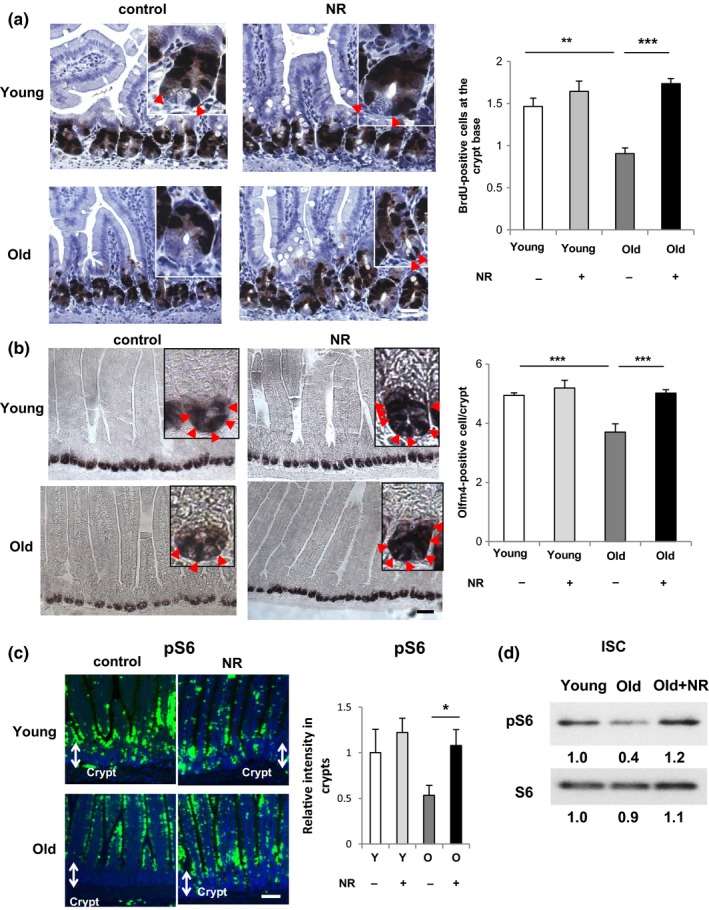
NR treatment restores ISC number in aged mice in vivo. (a) BrdU staining images and the quantification of BrdU‐positive CBC cells (arrowheads) in young or old mice administered with vehicle or NR (500 mg/kg) in drinking water for 6 weeks mice as assessed 2 hr after the injection of BrdU (3–5 mice per group, 50 intact well‐orientated crypts per mouse). (b) In situ hybridization images of Olfm4 mRNA and the quantification of Olfm4+ ISCs (arrowheads) in young or old mice administered with vehicle or NR (500 mg/kg) in drinking water for 6 weeks (eight mice per group, 50 intact well‐orientated crypts per mouse). (c) In situ phospho S6 (pS6) staining images (green, pS6; blue, DAPI) in the intestine of young or old mice administered with vehicle or NR (500 mg/kg) in drinking water for 6 weeks and the quantification of relative signal intensities per area in crypt (five mice per group). At least four different image fields per each sample were quantified by Image J. Y:Young, O:Old. Original magnifications: ×400 (a); ×200 (b); and ×100 (c). Scale bar: 25 µm (a); 50 µm (b); and 100 µm (c). (d) Immunoblotting of pS6 or S6 in ISCs isolated from young or old *Lgr5-EGFP-IRES-CreERT2* mice administered with vehicle or NR. Values represent the mean ± *SEM*. *p < 0.05; **p < 0.01; ***p < 0.001; *t* test, CBC: crypt base columnar; ISCs: intestinal stem cells; NR: nicotinamide riboside

We wished to test whether the decline in ISC number in old mice could be reversed by NAD^+^ replenishment. Thus, we tested whether NR treatment affects the number of ISCs in vivo in young or old mice with a 6 weeks treatment of drinking water containing NR (500 mg/kg body weight) or vehicle (Figure 4).

First, we tested whether NAD^+^ supplementation affects the function of ISCs derived from old mice, but not Paneth cells in vivo. In vivo, NR treatment completely rescued the diminishment in colony formation efficiency in old crypts and old ISCs (Supporting Information Figure S5a–c). On the other hand, by combining ISCs and Paneth cells purified from young or old mice, we determined that NR treatment affects ISCs rather than Paneth cells (Supporting Information Figure S5d).

Next, we observed that the elongation of villi induced during aging was abrogated by NR treatment (Supporting Information Figure S6a). To investigate how NR treatment influenced the activity and number of ISCs in young and old mice, BrdU labeling and ISH for the ISC marker Olfm4 were performed. NR treatment completely rescued the decrease of BrdU uptake into CBC cells (Figure 4a) and, more importantly, the decrease of Olfm4+‐positive cells (Figure 4b). Again, NR treatment had minimal effects on the expansion of ISCs in young mice (Figure 4a,b). Moreover, we found a significant increase in SIRT1 protein and S6 phosphorylation in NR‐treated old crypts and old ISCs, confirming that ISC functional improvement by in vivo NAD^+^ replenishment activates the SIRT1/mTORC1 signaling pathway (Figure 4c,d and Supporting Information Figure S6b).

### 2.5 NR rescues functional defects in aging mouse gut

Finally, we wished to determine whether the reduction in ISC number would correspond to functional defects in the aging gut. The colon is sensitive to dextran sulfate sodium (DSS), which causes injury requiring tissue regeneration by colonic crypts (Yui et al., 2012; Zhao et al., 2015). Therefore, DSS colitis is a good model for investigating the regenerative function of ISCs.

**Figure 5:**
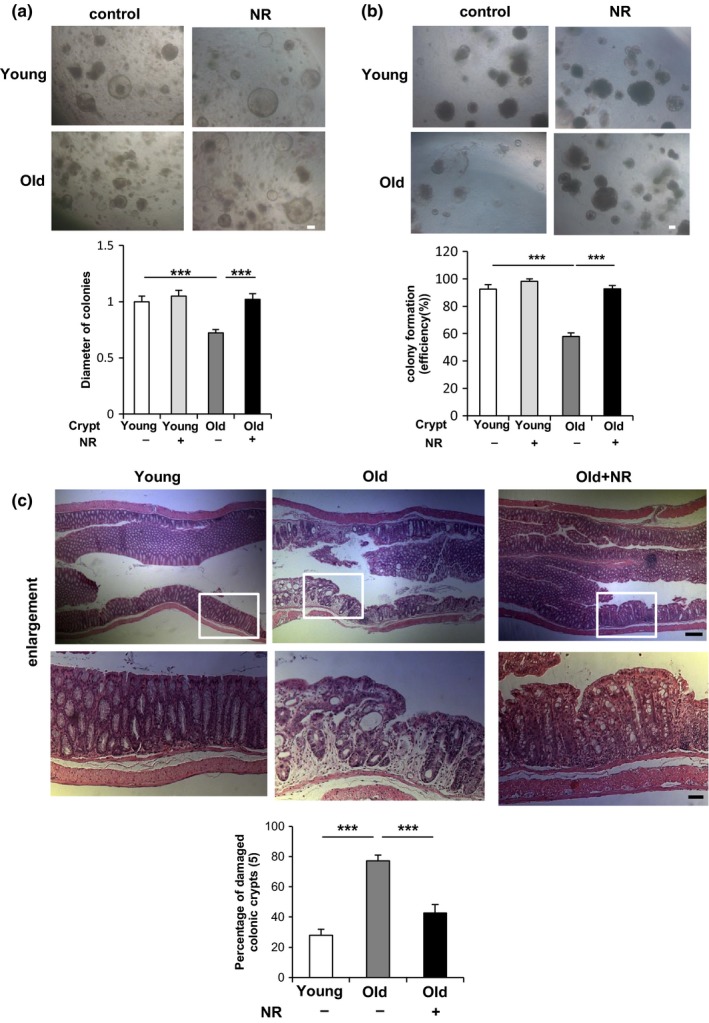
NR treatment reverses functional decline in ISCs during aging in vivo. (a) Isolated young or old colonic crypts were cultured in medium with or without 1 mM NR as indicated. The diameter of more than 70 colonies per each group was analyzed on day 5. (b) The number of colonies cultured in medium with or without 1 mM NR was assessed after 6 days of primary culture and an additional 5 days of secondary passage (4–5 wells per group). Original magnifications: ×50 (a and b). Scale bar: 100 µm (a and b). (c) Young and old mice administered with vehicle or NR (500 mg/kg) in drinking water for 6 weeks were treated by 1.5% DSS for 5 days followed by 3‐day water feeding before being sacrificed (five mice per group). The HE staining of distal colon (10 mm from middle) tissues and the percentage of damaged colonic crypts are shown. Bottom: the enlargement of the squared area. At least three different ×40 image fields per each sample were quantified. Original magnifications: ×50 and ×200 (enlargement). Scale bar: 200 and 50 µm (enlargement). Values represent the mean ± *SEM*. ***p < 0.001; *t* test. DSS: dextran sulfate sodium; ISCs: intestinal stem cells; NR: nicotinamide riboside

First, to investigate the effects of aging on the proliferation of colonic stem cells, we isolated crypts from colon of young or old mice and seeded them on Matrigel to obtain sphere colonies (O'Rourke, Ackerman, Dow, & Lowe, 2016). Colonic crypts from old mice formed as many but smaller colonies compared with young colonic crypts on day 5 (Figure 5a). Furthermore, colony formation efficiency from old colonic crypt significantly decreased after an additional 5 days of secondary passage (Figure 5b). NR treatment completely rescued those defects in colonies formed from old colonic crypts, while having a minimal effect in the colony formation from young colonic crypts (Figure 5a,b).

To test whether old mice were more sensitive to DSS, mice were administered 1.5% DSS for 5 days followed by 3‐day recovery periods before analysis. Old mice were found to be much more susceptible to DSS‐induced damage than young mice (Figure 5c). To test the ability of NR to rescue this deficit, young and old mice pretreated by NR or vehicle for 6 weeks before DSS challenge. Indeed, NR treatment suppressed the increase in susceptibility to DSS to levels observed in young mice (Figure 5c). The above data demonstrate that NR rescues damage repair defects in the aging gut, likely by restoring the ISC pool to numbers found in young mice.

## 3 DISCUSSION

The deterioration of organ integrity with aging can be in part attributed to a decline in adult stem cell number and function. Here we investigate the fate of ISCs in aging mice, both in terms of cell number and cell function. Our findings clearly show a decline in ISC cell number in 24 months or older mice. In contrast to ISCs, niche cells or Paneth cells showed no decline in cell number in old mice. One recent paper did not find a decline in ISC number in old mice (Nalapareddy et al., 2017), but another study (Mihaylova et al., 2018) did. This disparity may reflect different strains, housing conditions, or methods. Similarly, we assessed the abilities of ISCs and Paneth cells to function ex vivo by their ability to give rise to organoids in a Matrigel matrix. The organoid assay again revealed a functional defect in ISCs but not in Paneth cells from old mice.

Other studies have shown the benefits of NAD^+^ replenishment by the NAD^+^ precursors NR and NMN in metabolic function (Cantó et al., 2012; Yoshino, Mills, Yoon, & Imai, 2011) and stem cell number (Zhang et al., 2016), especially MuSCs. In addition, SIRT3 up‐regulation has been shown to rescue aging deficits in hematopoietic stem cells (Brown et al., 2013). The fact that we observed rescue of ISC number and function by NR expands our understanding of stem cell aging. Most interestingly, rapamycin blocked the rejuvenation of ISCs by NR. This is because SIRT1 and mTORC1 are both activated and function sequentially in response to the Paneth cell signal cADPR (Igarashi & Guarente, 2016). Indeed, SIRT1 deacetylates S6K1 fostering its phosphorylation by mTORC1 and the activation of translation accompanied by an increase in ISC number (Igarashi & Guarente, 2016). Our findings suggest that NAD^+^ replenishment may be a viable strategy for maintaining gut health with aging. Nalapareddy et al. (2017) and Mihaylova et al. (2018) also reported a decline in organoid formation in crypts derived from old mice, and this could be rescued by elevating WTN signaling (Nalapareddy et al., 2017) or activating a fatty acid oxidation program (Mihaylova et al., 2018). It is thus likely that multiple signaling pathways affecting ISCs can be triggered to rejuvenate the aging gut.

It is noteworthy that mitochondrial defects are a common thread in the decline of adult stem cells. Similarly, defects in DNA repair leading to premature aging also triggers NAD^+^ depletion via PARP and leads to defective mitochondria (Fang et al., 2014). In both aging stem cells and cells with DNA repair deficits, the mitochondrial defects can be rescued by NAD^+^ replenishment and SIRT1 activation. Along with our findings in the gut, these results all suggest that NAD^+^ replenishment may be a viable strategy to slow aging in a variety of tissues.

Our findings also raise the possibility that anti‐aging interventions, like rapamycin, may have beneficial consequences in some contexts but actually be deleterious in others. It will be interesting to see whether small molecule interventions (e.g., rapamycin, metformin, NR) show different tissue selectivity in their abilities to improve function during aging. It is possible that different individuals will respond best to one or another intervention, depending on which organ is most vulnerable to aging in that person. Human trials over the next few years may shed light on this important question.

## 4 EXPERIMENTAL PROCEDURES

### 4.1 Materials and Methods

#### 4.1.1 Mice

Aged (more than 24 months old) male C57BL/6 mice and young (3–5 months old) control mice were obtained from the National Institute of Aging mouse colony or Japan SLC Inc (Shizuoka, Japan). Lgr5‐EGFP‐IRES‐CreERT2 mice were purchased from Jackson Laboratories. All mice were maintained in the C57BL/6 background and were housed on a 12:12‐hr light:dark cycle at controlled temperature (25 ± 1°C). All animal procedures were in accordance with the animal care committee of MIT and the University of Tokyo.

#### 4.1.2 Nicotinamide riboside treatment

Mice were administered with NR (ChromaDex) at a concentration of 500 mg kg^−1^ day^−1^ in drinking water for 6 weeks. Water bottles were exchanged every day.

#### 4.1.3 Dextran sodium sulfate treatment

Nicotinamide riboside (500 mg kg^−1^ day^−1^ for 6 weeks) or vehicle‐treated mice were subjected to 1.5% DSS with or without NR (500 mg kg^−1^ day^−1^) dissolved in drinking water for 5 days. Additional intraperitoneal injection of NR (1,000 mg kg^−1^ day^−1^) or vehicle was employed during 5 day DSS treatment. At day 8 after the start of DSS treatment, the distal colon (10 mm from middle) was isolated and used for HE staining.

#### 4.1.4 Immunohistochemistry

Pieces of the proximal jejunum (1–4 cm from pylorus) were fixed overnight in 10% neutral‐buffered formalin at room temperature, embedded in paraffin, and sectioned. Sections were deparaffinized and subjected to antigen retrieval with 10 mM sodium citrate (pH 6.0) in a 95°C water bath for 40 min. Slides were then incubated with the primary antibodies overnight at 4°C. The primary antibodies used were rat anti‐BrdU (1/200; Abcam 6326), rabbit anti‐lysozyme (1/50; Thermo Scientific PA5‐16668), goat anti‐chromogranin A (1/50; Santa Cruz sc‐1488), and rabbit anti‐phosphoS6 Ser235/236 (1/400, Cell Signaling 4858). Further staining steps were carried out with TSA Plus Cyanine 3 System (PerkinElmer) according to the manufacturer's instructions. Finally, slides were mounted in Vectashield Mounting Medium with DAPI (Vector). For the staining of BrdU and phospho‐S6, Biotin‐conjugated secondary antibody was used, followed by the Vectastain Elite ABC immunoperoxidase detection kit (Vector) and Dako Liquid DAB+ Substrate (DAKO) for visualization. Microscopic images were obtained by a Zeiss Axio Imager M1 fluorescent microscope or Keyence All‐in‐One Fluorescence Microscope (BZ‐X710), and the fluorescent intensities were quantified by ImageJ software.

#### 4.1.5 In situ hybridization

Digoxigenin (DIG)‐labeled Olfm4 RNA probe was made using cDNA (IMAGE mouse cDNA clone 9055739) obtained from GE Healthcare as templates. To confirm the specificity of the probe, we generated both sense and antisense probe. Detailed methods are described previously (Gregorieff & Clevers, 2010).

#### 4.1.6 Quantification

Crypt length and villus length were measured from the bottom of the crypt to the crypt–villus junction and from the crypt–villus junction to the tip of the villus, respectively, by ImageJ software. Quantification in each mouse was performed from 50–150 crypt/villus units per mouse. BrdU‐positive CBC cells were counted as BrdU‐positive cells adjacent to Paneth cells visualized at the bottom of crypts. All Olfm4‐positive cells, which positions from 0 to +4 in crypt, were counted.

Quantification of BrdU orOlfm4‐positive cells in each mouse was performed from approximately 50 intact, well‐orientated crypts per mouse under Zeiss Axio Imager M1 microscope. The number of mice analyzed is indicated in figure legends.

The diameter of formed organoid colonies was measured by ImageJ software.

#### 4.1.7 Crypt isolation and culture

Crypts were isolated from small intestine described previously (Sato & Clevers, 2013; Sato et al., 2009). Briefly, the proximal half (duodenum and upper half of jejunum) of mouse small intestine was isolated, opened longitudinally, and washed with cold PBS. We then scraped the intestine using a coverslip and removed the villi. After washing the intestine with cold PBS, we cut the intestine into small (2–4 mm) pieces with scissors and washed them in cold PBS, pipetting up and down 5–10 times with a pipette. Washed pieces of the intestine were gently rocked in PBS containing 2 mM EDTA for 30 min at 4°C. Next, we pipetted pieces of the intestine up and down in cold PBS with a pipette until most of crypts are released. We got the crypt fraction by passing the suspension through a 70‐µm cell strainer (Corning) and centrifuging at 250 *g* for 5 min.

Isolated crypts were collected in crypt culture medium, counted, and embedded in Matrigel (Corning) at 10 crypts/μl. A total of 300 isolated crypts were plated per well of a 48‐well plate and cultured in a crypt culture medium, DMEM/F12 (Thermo Scientific) supplemented by 1× N2 (Thermo Scientific), 1× B27 (Thermo Scientific), 1 mM N‐Acetyl‐L‐cysteine (Sigma), 50 ng/ml EGF (Thermo Scientific), 100 ng/ml Noggin (Peprotech), and 500 ng/ml R‐spondin (R&D or ACRO Biosystems). The medium was changed every other day. When indicated, NR (ChromaDex), rapamycin (Santa cruz) or EX527 (Santa cruz) was added to crypt culture medium. The number of organoids and the number per organoid of differentiated buds were counted 5 days after plating. In Figure 2, the absolute value of organoids is shown in *y*‐axis. In the other figures, the ratio to control is shown in *y*‐axis.

#### 4.1.8 Colonic crypt isolation and culture

Colonic crypts were isolated from large intestine described previously (O'Rourke et al., 2016). Briefly, 5–7 cm or the proximal large intestine was isolated, opened longitudinally, and washed with cold PBS. Using a glass slide, the lumen of the intestine was scraped to remove fecal matter and mucosa. After washing the intestine with cold PBS, we cut the intestine into small (5 mm) pieces with scissors and placed into cold 5 mM EDTA‐PBS. Pieces of the intestine were gently rocked for 15 min at 4°C. After removal of EDTA‐PBS, pieces of the intestine were incubated in the medium containing 500 U/ml Collagenase Type IV for 30 min at in 37°C water bath. Next, we pipetted pieces of the intestine up and down in cold PBS with a pipette until most of crypts are released. We got the crypt fraction by passing the suspension through a 70‐µm cell strainer and centrifuging at 250 *g* for 5 min.

Isolated crypts were collected in crypt culture medium, counted, and embedded in Matrigel. A total of 1,000 isolated crypts were plated per well of a 48‐well plate and cultured in a colonic crypt culture medium, DMEM/F12 supplemented by 1× N2, 1× B27, 1 mM N‐Acetyl‐L‐cysteine, 50 ng/ml EGF, 100 ng/ml Noggin, 500 ng/ml R‐spondin, 2 mM Valprorc acid (Wako), and 10 µM CHIR99021 (Stemgent). The medium was changed every other day. When indicated, NR was added to crypt culture medium. The diameter of formed sphere organoids was measured 5 days after plating. For secondary organoid assays, primary organoids were passaged as described previously (O'Rourke et al., 2016) on the sixth day after initial plating.. The percentages of formed organoid were calculated based on the number of enterospheres observed on day 1 after passaging.

#### 4.1.9 Flow cytometry

Intestinal stem cells and Paneth cell isolation was performed as described previously (Igarashi & Guarente, 2016; Roth et al., 2012: Yilmaz et al., 2012). Briefly, the crypt suspensions were centrifuged for 5 min at 250 *g* at 4°C. The pellets were gently resuspended in 1.0 ml of undiluted TrypLE Express (Life Technologies) +120 μl of DNase I (10 U/μl, Roche). The suspended crypts were incubated in a 32°C water bath for 1.5 min were then placed on ice. 12 ml of cold MEM was added, and crypts were gently triturated twice. After centrifugation, the pellets were resuspended and incubated for 15 min on ice in 1 ml MEM containing CD45‐PE (1/500 eBioscience), CD31‐PE (1/500 Biolegend), Ter119‐PE (1/500 Biolegend), and CD24‐Pacific Blue (1/500 Biolegend). After centrifugation, the pellets were resuspended with MEM containing 1.5 μM propidium iodide (PI) (Thermo Scientific). The samples were filtered through a 40‐μm mesh (Corning) and immediately sorted on a FACS Aria (Becton Dickinson).

Intestinal stem cells were isolated as Lgr5‐EGFP^hi^CD24^low/−^CD31^−^Ter119^−^CD45^−^PI^−^,and Paneth cells were isolated as CD24^hi^SideScatter^hi^LgR5‐EGFP^−^CD31^−^Ter119^−^CD45^−^PI^−^ (Igarashi & Guarente, 2016).

#### 4.1.10 Culture of isolated ISCs and Paneth cells

Isolated ISCs or Paneth cells were suspended in the medium containing 1× N2, 1× B27, and 10 µM Y‐27632 (Sigma). ISCs 2,000 cells with or without Paneth cells 2,000 cells were then seeded into 30 µl Matrigel containing 1 µM Jagged‐1 (AnaSpec) and 10 µM Y‐27632. The Matrigel drops with ISCs with or without Paneth cells were allowed to solidify on a 48‐well plate for 15 min in a 37°C incubator. The culture medium containing 1× N2, 1× B27, 1 mM N‐Acetyl‐L‐cysteine, 50 ng/ml EGF, 100 ng/ml Noggin, 1 µg/ml R‐Spondin (ACRO Biosystems) (0.5 µg/ml R‐Spondin (R&D) with 10 µM CHIR99021) was then overlayed onto the drops of Matrigel and maintained at 37°C incubator. When indicated, NR (ChromaDex), rapamycin (Santa cruz) or EX527 (Santa cruz) was added to the culture medium. The medium was changed every 2 days. The number of colonies with lumen and the diameter of colonies were quantitated at day 5 of culture. In Supporting Information Figure S5c, ISCs were cultured without CHIR99021 after day 4 and formed organoids (not spheres) were assessed at day 9 of culture. In Figure 2, the absolute value of organoids is shown in *y*‐axis. In the other figures, the ratio to control is shown in *y*‐axis.

#### 4.1.11 Immunoblotting

Antibodies for immunoblotting were obtained from the following sources: rabbit anti‐GAPDH (Cell signaling 5174), rabbit anti‐SIRT1 (Cell Signaling 2028), rabbit anti‐phospho‐S6 Ser235/236 (Cell Signaling 4858), and rabbit anti‐S6 (Cell Signaling 2217). Isolated intestine, crypts, or ISCs were lysed in RIPA buffer supplemented with protease Inhibitor and phosphatase inhibitor (Santa Cruz). Proteins extracts were denatured by the addition of SDS loading buffer, boiled and resolved by SDS‐PAGE, and analyzed by immunoblotting with primary antibodies listed above. The band density of all blots was quantified by ImageJ software.

## CONFLICT OF INTEREST

LG is a founder of Elysium Health and Galelei BioSciences and consults for Sibelius, and Segterra.
