# Supplementary material for: NAD+ supplementation rejuvenates aged gut adult stem cells
Source: Aging Cell. 2019 Mar 27;18(3):e12935. doi: 10.1111/acel.12935 (PMC6516145; doi:10.1111/acel.12935)
Supplement: Supplementary file 1 [file ACEL-18-e12935-s001.pdf]

# Figure S1

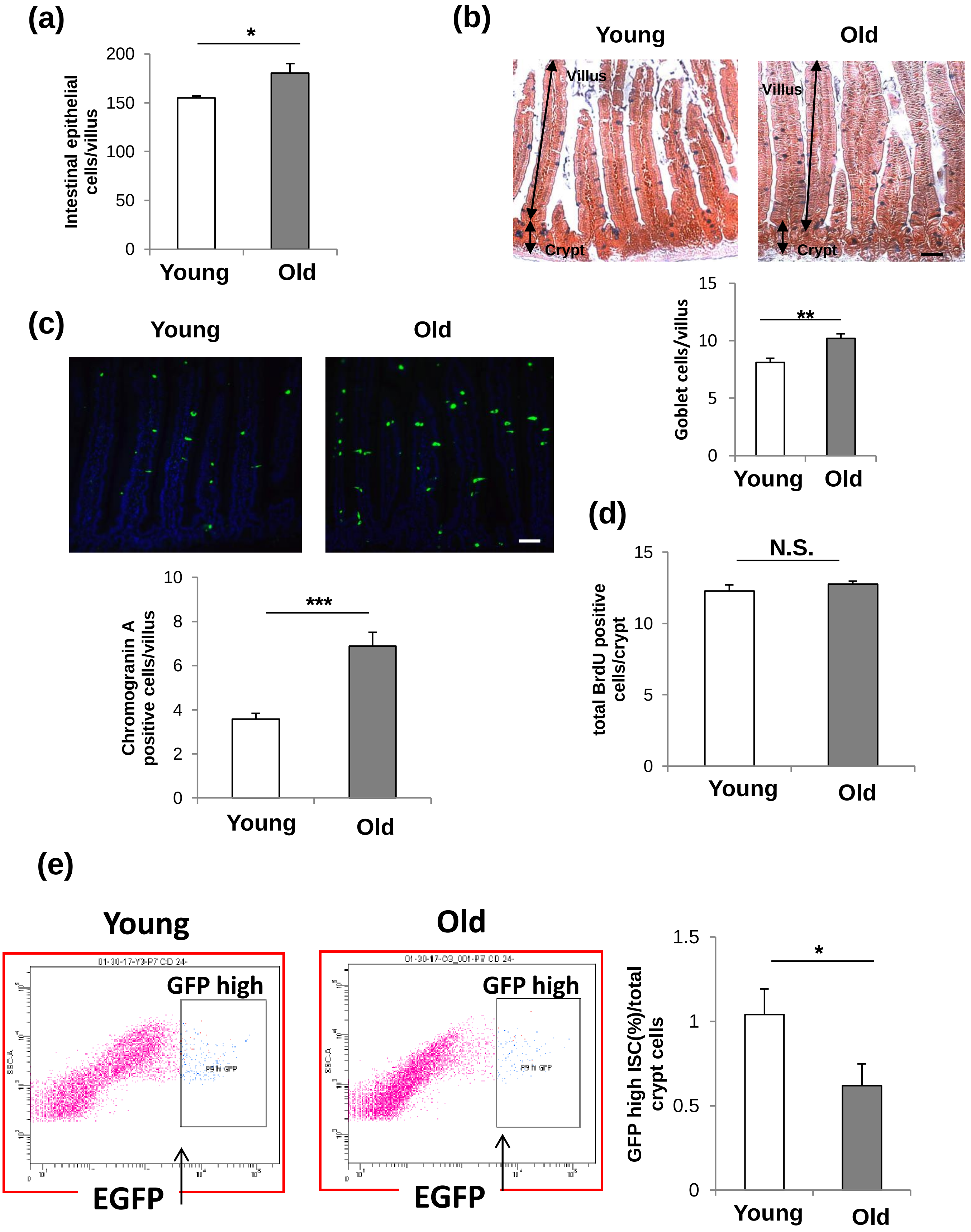
(a)
*
200
150
100
50
0
Intestinal epithelial cells/villus
Young
Old
(b)
Young
Old
Villus
Crypt
15
10
5
0
**
Goblet cells/villus
Young
Old
(c)
Young
Old
10
8
6
4
2
0
***
Chromogranin A positive cells/villus
Young
Old
(d)
N.S.
15
10
5
0
total BrdU positive cells/crypt
Young
Old
(e)
Young
Old
GFP high
GFP high
EGFP
EGFP
1.5
1
0.5
0
*
GFP high ISC(%)/total crypt cells
Young
Old

# Figure S2

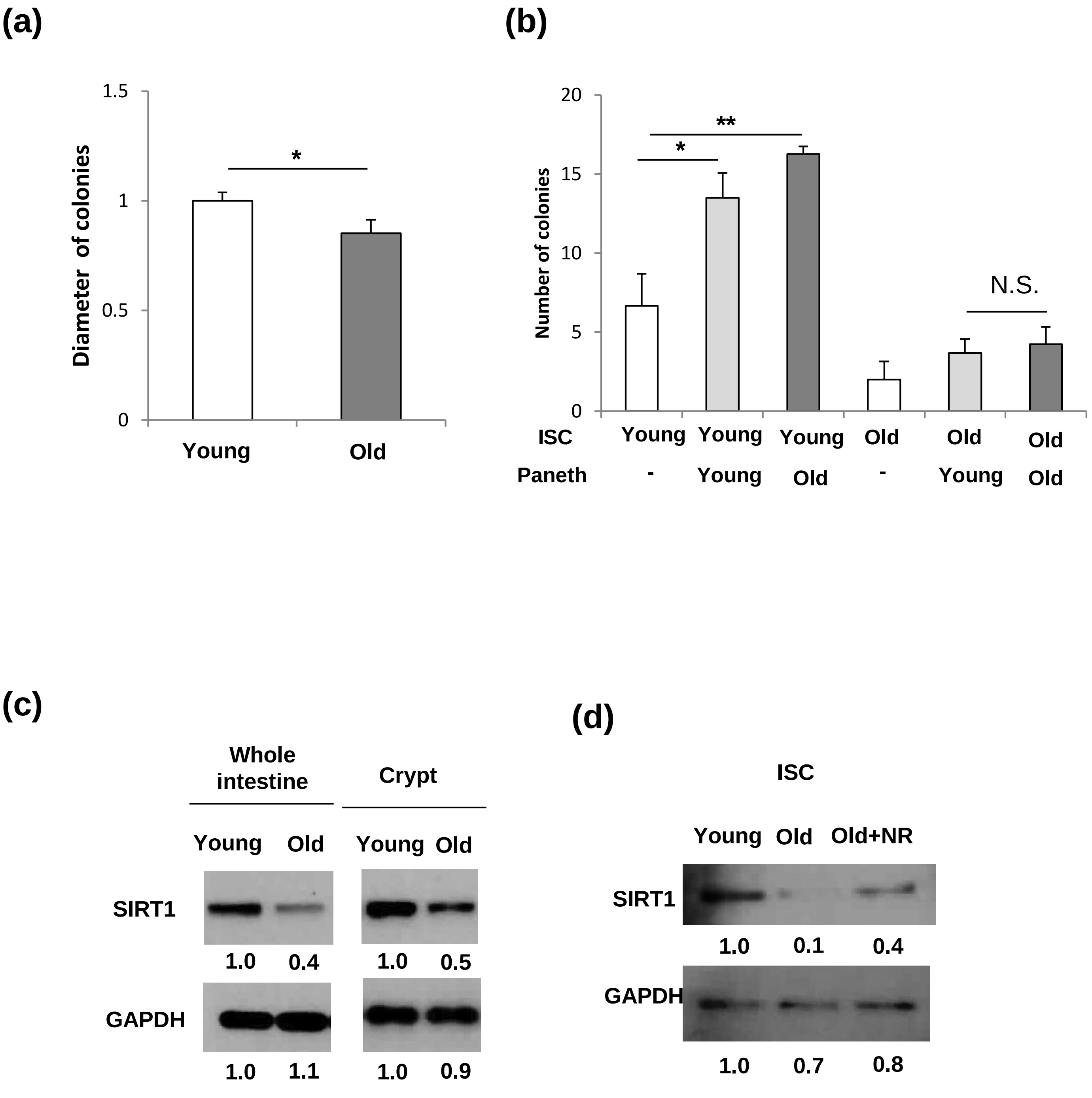

(a)
Diameter of colonies
1.5
1
0.5
0
*
Young
Old
(b)
Number of colonies
20
15
10
5
0
**
*
N.S.
ISC
Young
Young
Young
Old
Old
Old
Paneth
-
Young
Old
-
Young
Old
(c)
Whole intestine
Crypt
Young
Old
Young
Old
SIRT1
1.0
0.4
1.0
0.5
GAPDH
1.0
1.1
1.0
0.9
(d)
ISC
Young
Old
Old+NR
SIRT1
1.0
0.1
0.4
GAPDH
1.0
0.7
0.8

# Figure S3

## (a)

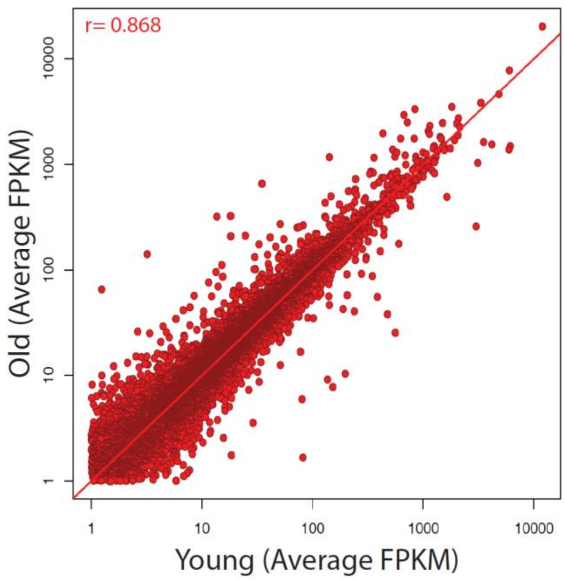


## (b)

| Gene ID | Young (Average FPKM) | Old (Average FPKM) | log2(fold_change) | q_value |
|---|---|---|---|---|
| Reg3b | 670.052 | 2943.42 | 2.13515 | 0.00910147 |
| Apoa1 | 715.457 | 2490.49 | 1.79949 | 0.00910147 |
| Mir5121,Rpl13a,Snord33 | 1146.72 | 2321.22 | 1.01736 | 0.0350321 |
| Apoa4 | 620.431 | 1768.78 | 1.51141 | 0.0430539 |
| Fabp1 | 826.531 | 1768.72 | 1.09757 | 0.00910147 |
| Rbp2 | 526.281 | 1377.22 | 1.38786 | 0.00910147 |
| 2210404O07Rik | 554.189 | 1187.14 | 1.09904 | 0.0215895 |
| 2200002D01Rik | 336.296 | 855.33 | 1.34675 | 0.00910147 |
| Rabggtb,Snord45c | 34.8038 | 655.257 | 4.23474 | 0.00910147 |
| Mt1 | 242.162 | 580.198 | 1.26057 | 0.00910147 |
| Guca2b | 129.707 | 433.729 | 1.74154 | 0.00910147 |
| Hipk3,Mir1902 | 13.6118 | 319.294 | 4.55196 | 0.00910147 |
| Sis | 72.2196 | 252.402 | 1.80526 | 0.00910147 |
| Bpnt1 | 109.886 | 220.255 | 1.00317 | 0.0315765 |
| Slc9a3r1 | 106.041 | 219.805 | 1.0516 | 0.0315765 |
| Mt2 | 24.5766 | 213.1 | 3.11617 | 0.00910147 |
| Coro2a | 91.6118 | 206.147 | 1.17007 | 0.00910147 |
| Slc5a1 | 78.0694 | 186.067 | 1.25299 | 0.0215895 |
| Gstm1 | 46.2149 | 166.077 | 1.84542 | 0.00910147 |
| Apob | 51.2973 | 144.219 | 1.49131 | 0.0215895 |
| Papss2 | 49.3341 | 133.235 | 1.43332 | 0.00910147 |
| Kcne3 | 303.848 | 132.579 | -1.1965 | 0.0275067 |
| Enpep | 49.3548 | 129.981 | 1.39704 | 0.00910147 |
| Ifitm3 | 303.254 | 125.733 | -1.27016 | 0.0154725 |
| Aldh1a1 | 53.1201 | 120.043 | 1.17622 | 0.00910147 |
| Acadl | 60.4367 | 119.126 | 0.978994 | 0.0215895 |
| Sepp1 | 39.6199 | 114.424 | 1.5301 | 0.00910147 |
| Ces1f | 50.0747 | 106.207 | 1.08473 | 0.0350321 |
| Gpd1 | 49.1049 | 102.752 | 1.06523 | 0.0154725 |
| Creb3l3 | 30.3965 | 102.534 | 1.75413 | 0.00910147 |
| Ace | 29.6539 | 102.433 | 1.78839 | 0.00910147 |
| Slc6a6 | 40.6761 | 101.389 | 1.31765 | 0.00910147 |
| Clca3 | 275.156 | 92.0953 | -1.57905 | 0.00910147 |
| Mia2 | 40.8711 | 91.7703 | 1.16695 | 0.00910147 |
| Ms4a10 | 34.8073 | 91.4897 | 1.39422 | 0.0154725 |
| Akr1b7 | 15.3488 | 86.3315 | 2.49176 | 0.00910147 |
| Cyp4v3 | 33.814 | 84.8475 | 1.32725 | 0.00910147 |
| Akr1c19 | 32.7456 | 83.3349 | 1.34762 | 0.00910147 |
| Por | 39.1708 | 81.4973 | 1.05697 | 0.0350321 |
| Zfp36 | 36.0281 | 80.6137 | 1.1619 | 0.00910147 |
| Slc7a8 | 39.5007 | 77.4992 | 0.972303 | 0.0430539 |
| Ano6 | 34.0261 | 75.2988 | 1.14599 | 0.0154725 |
| Xdh | 30.8066 | 72.9073 | 1.24282 | 0.0154725 |
| Ace2 | 29.3893 | 69.9477 | 1.25098 | 0.0154725 |
| Cyp3a11 | 19.3146 | 63.7501 | 1.72273 | 0.00910147 |
| Gpt | 19.4496 | 63.0328 | 1.69636 | 0.00910147 |
| Npc1l1 | 30.8624 | 62.98 | 1.02904 | 0.0460339 |
| Cyp2c65 | 24.5379 | 62.8853 | 1.35771 | 0.00910147 |
| Cdr2 | 21.4597 | 62.8084 | 1.54932 | 0.00910147 |
| Tgfbi | 26.9651 | 59.899 | 1.15144 | 0.0350321 |
| Vat1 | 21.8065 | 59.4886 | 1.44785 | 0.00910147 |
| Inpp5b,Mir698 | 20.6095 | 59.1636 | 1.5214 | 0.00910147 |
| Cobl | 18.9471 | 58.0816 | 1.6161 | 0.00910147 |
| Ang4 | 204.753 | 57.9512 | -1.82097 | 0.00910147 |
| Cited2 | 25.6471 | 57.3092 | 1.15997 | 0.0275067 |
| Pck1 | 8.48364 | 57.0149 | 2.74858 | 0.00910147 |
| Fkbp5 | 12.1961 | 56.6774 | 2.21636 | 0.00910147 |
| Axin2 | 112.343 | 56.2103 | -0.999006 | 0.0499113 |

| Gene ID | Young (Average FPKM) | Old (Average FPKM) | log2(fold_change) | q_value |
|---|---|---|---|---|
| Hsph1 | 139.538 | 56.1016 | -1.31454 | 0.0460339 |
| Lgals3 | 23.8038 | 55.0113 | 1.20854 | 0.0350321 |
| Ggt1 | 20.1541 | 53.2701 | 1.40225 | 0.00910147 |
| Cyp2b10 | 15.9021 | 48.4649 | 1.60772 | 0.00910147 |
| Cyp3a13 | 19.4557 | 47.2981 | 1.28159 | 0.00910147 |
| Hpgd | 19.875 | 47.2711 | 1.25001 | 0.0154725 |
| Cyp2d26 | 18.1035 | 45.3326 | 1.32428 | 0.0315765 |
| Mir17hg,Mir19b-1 | 7.76562 | 44.5232 | 2.51939 | 0.0460339 |
| Abcc2 | 14.7825 | 41.405 | 1.48591 | 0.00910147 |
| Nr1d1 | 10.3352 | 40.6452 | 1.97552 | 0.00910147 |
| Slc26a3 | 18.0152 | 40.1854 | 1.15746 | 0.0315765 |
| Fbp1 | 13.8486 | 40.1738 | 1.53651 | 0.0315765 |
| Slc25a24 | 15.3096 | 38.3472 | 1.32469 | 0.00910147 |
| Mme | 18.6712 | 37.3987 | 1.00217 | 0.0393845 |
| Hsd17b13 | 10.217 | 35.6409 | 1.80256 | 0.0499113 |
| Cirbp | 5.81019 | 34.6005 | 2.57414 | 0.0154725 |
| Slc6a8 | 16.0509 | 34.216 | 1.09202 | 0.0430539 |
| Abcg8 | 13.2486 | 32.2835 | 1.28496 | 0.0154725 |
| Dgat2 | 11.3642 | 31.911 | 1.48956 | 0.0215895 |
| Maf | 14.0848 | 31.7493 | 1.17259 | 0.0350321 |
| Fmo5 | 15.0333 | 31.0948 | 1.04851 | 0.0275067 |
| Slc2a2 | 7.74252 | 30.0048 | 1.95432 | 0.00910147 |
| Mir3062,Pfas | 12.7225 | 29.4883 | 1.21277 | 0.0393845 |
| Akr1b8 | 4.44455 | 29.4738 | 2.72932 | 0.0315765 |
| Slc15a1 | 8.97078 | 27.1441 | 1.59733 | 0.00910147 |
| Myc | 60.2757 | 25.8612 | -1.22079 | 0.00910147 |
| Iyd | 3.36181 | 25.1313 | 2.90218 | 0.0154725 |
| Sec24d | 10.8971 | 24.7369 | 1.18273 | 0.0350321 |
| Cyp27a1 | 6.87583 | 23.9324 | 1.79936 | 0.0430539 |
| Slc6a19 | 5.07631 | 23.0961 | 2.1858 | 0.00910147 |
| Prelp | 52.0403 | 22.0911 | -1.23616 | 0.0460339 |
| Slfn9 | 50.3459 | 20.8801 | -1.26974 | 0.00910147 |
| Clec2e | 6.5875 | 20.6252 | 1.64661 | 0.0315765 |
| C530008M17Rik | 7.12146 | 17.7403 | 1.31679 | 0.00910147 |
| Mical2 | 8.19329 | 17.0683 | 1.0588 | 0.0460339 |
| Per1 | 5.54937 | 16.4541 | 1.56805 | 0.00910147 |
| Sowaha | 6.11869 | 15.9557 | 1.38277 | 0.0499113 |
| Slc2a5 | 4.6139 | 15.7766 | 1.77372 | 0.0315765 |
| Kif21b | 6.27432 | 15.7488 | 1.32771 | 0.00910147 |
| Tef | 2.5276 | 15.1986 | 2.5881 | 0.00910147 |
| Sgk1 | 2.81979 | 15.0906 | 2.41999 | 0.0460339 |
| Mafb | 3.25584 | 12.7761 | 1.97234 | 0.00910147 |
| Cd44 | 27.7856 | 11.2394 | -1.30578 | 0.0154725 |
| Clca2 | 25.7568 | 10.201 | -1.33623 | 0.0215895 |
| Zc3h6 | 2.21017 | 9.23341 | 2.06271 | 0.00910147 |
| Dnaja1,Mir207 | 136.43 | 9.13623 | -3.90042 | 0.00910147 |
| Cd36 | 1.85075 | 8.24773 | 2.15589 | 0.0393845 |
| Mir702,Plod3 | 80.1594 | 5.96569 | -3.74811 | 0.00910147 |
| Banp | 11.7527 | 3.60826 | -1.70362 | 0.0275067 |
| Gsdmc4 | 28.8604 | 3.58221 | -3.01017 | 0.00910147 |
| Adamts15 | 8.21375 | 2.96564 | -1.4697 | 0.0430539 |
| Mir678,Prmt2 | 81.6631 | 1.68033 | -5.60287 | 0.00910147 |

# Figure S4

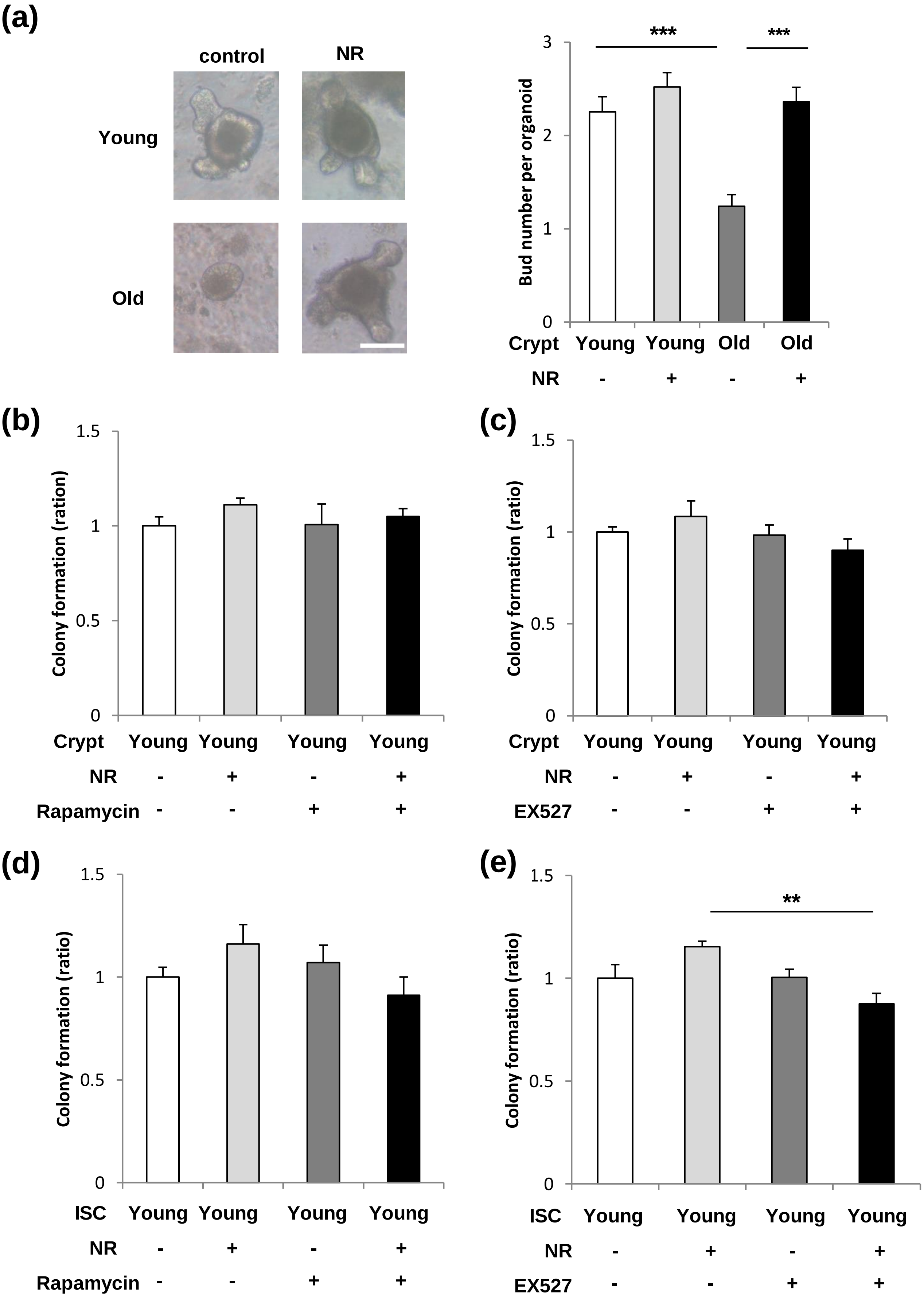
(a)
control
NR
Young
Old
***
***
Bud number per organoid
3
2
1
0
Crypt Young Young Old Old
NR - + - +
(b)
Colony formation (ration)
1.5
1
0.5
0
Crypt Young Young Young Young
NR - + - +
Rapamycin - - + +
(c)
Colony formation (ratio)
1.5
1
0.5
0
Crypt Young Young Young Young
NR - + - +
EX527 - - + +
(d)
Colony formation (ratio)
1.5
1
0.5
0
ISC Young Young Young Young
NR - + - +
Rapamycin - - + +
(e)
**
Colony formation (ratio)
1.5
1
0.5
0
ISC Young Young Young Young
NR - + - +
EX527 - - + +

# Figure S5

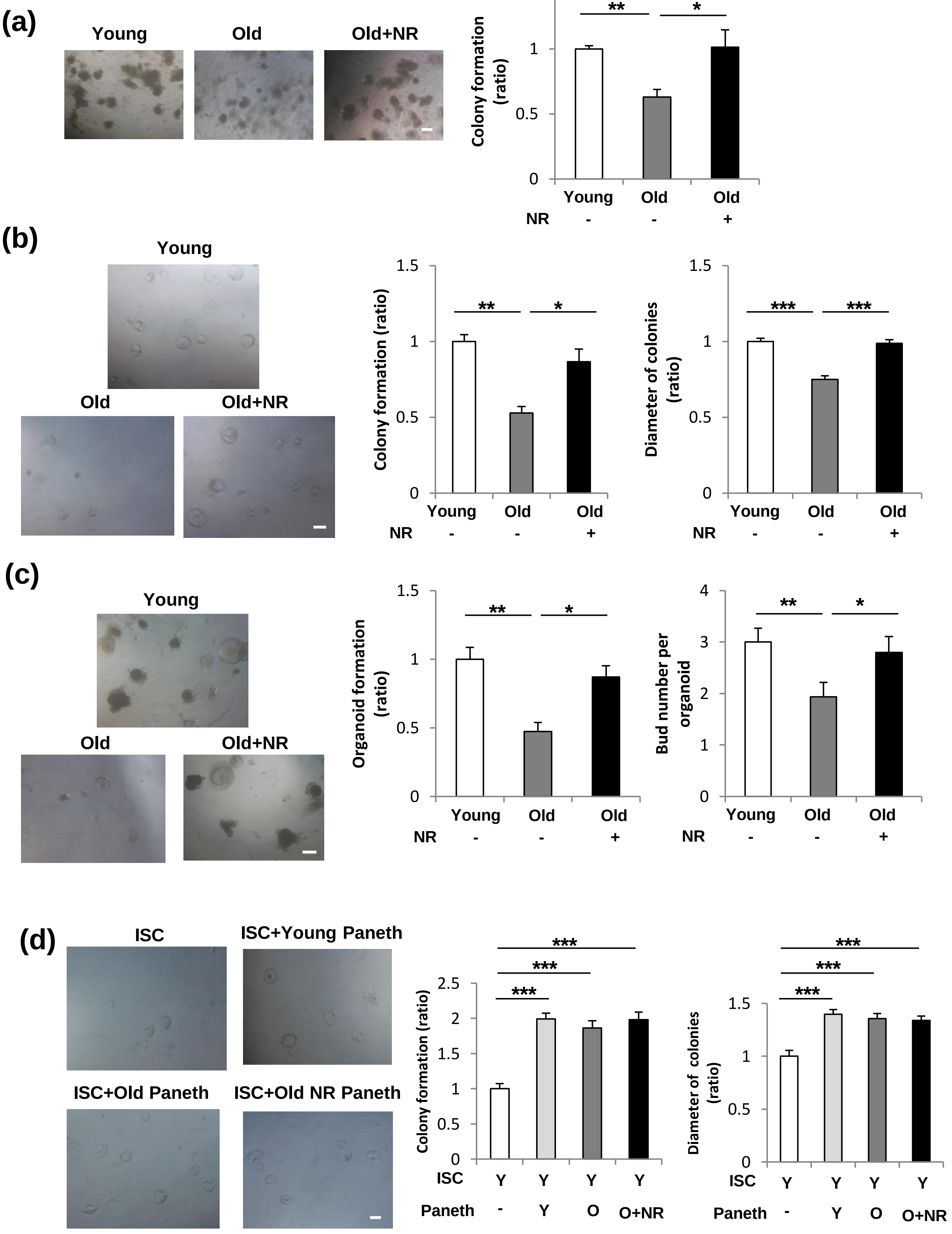

(a)
Young
Old
Old+NR
Colony formation (ratio)
1.5
1
0.5
0
**
*
Young
Old
Old
NR
-
-
+
(b)
Young
Old
Old+NR
Colony formation (ratio)
Diameter of colonies (ratio)
**
*
***
***
Young
Old
Old
NR
-
-
+
(c)
Young
Old
Old+NR
Organoid formation (ratio)
Bud number per organoid
4
3
2
1
0
**
*
Young
Old
Old
NR
-
-
+
(d)
ISC
ISC+Young Paneth
ISC+Old Paneth
ISC+Old NR Paneth
Colony formation (ratio)
Diameter of colonies (ratio)
2.5
2
1.5
1
0.5
0
***
ISC
Y
Y
Y
Y
Paneth
-
Y
O
O+NR

# Figure S6

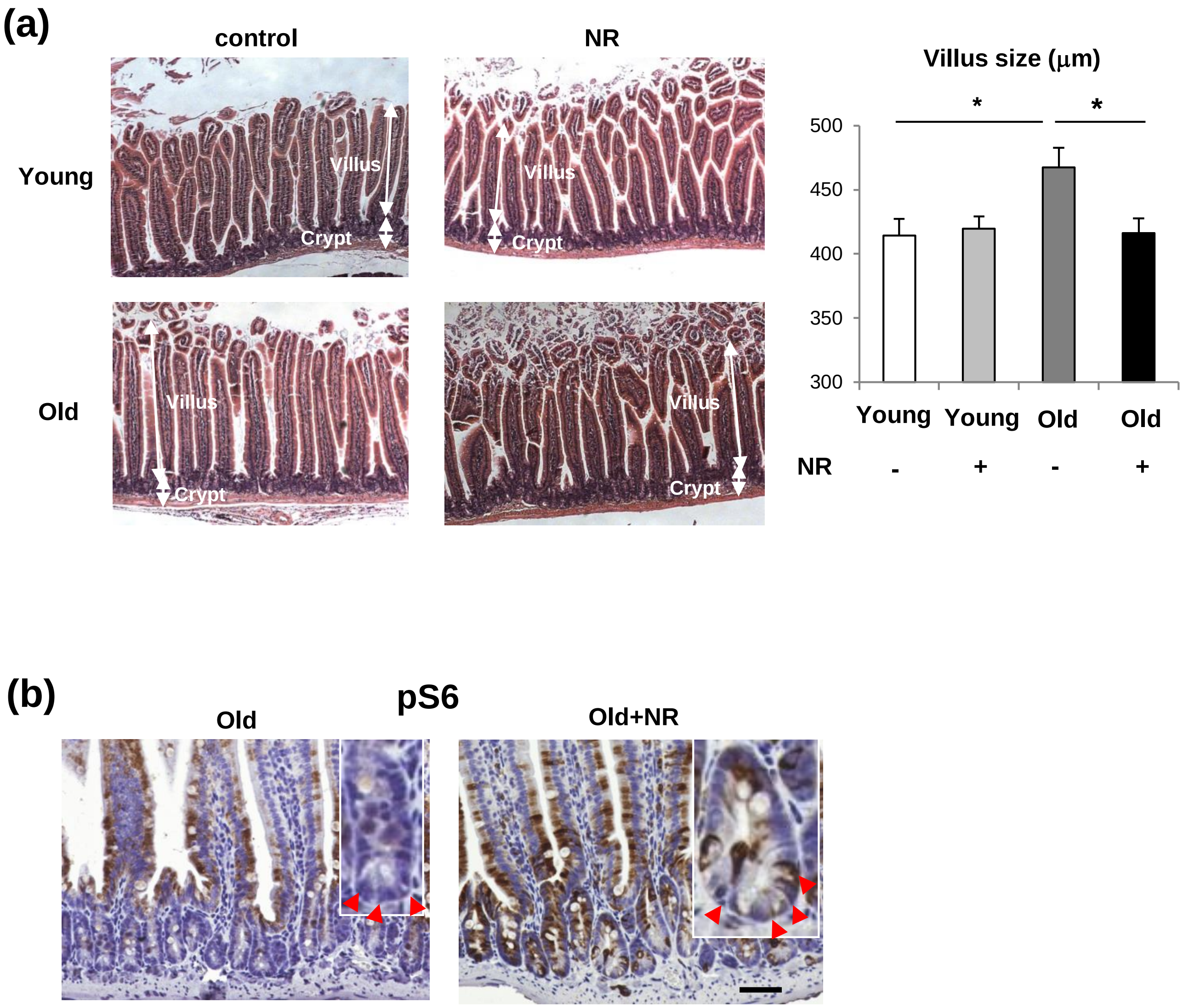
(a)
control
NR
Young
Old
Villus
Crypt
Villus size (μm)
*
*
500
450
400
350
300
Young
Young
Old
Old
NR
-
+
-
+
(b)
pS6
Old
Old+NR
