# Supplementary material for: NAD+ supplementation rejuvenates aged gut adult stem cells
Source: Aging Cell. 2019 Mar 27;18(3):e12935. doi: 10.1111/acel.12935 (PMC6516145; doi:10.1111/acel.12935)
Supplement: Supplementary file 2 [file ACEL-18-e12935-s002.docx]

**NAD+ supplementation rejuvenates aged gut adult stem cells.**

**Masaki Igarashi^1^, Masaomi Miura^1^, Eric Williams^2^ Frank Jaksch^3^, Takashi Kadowaki^1^, Toshimasa Yamauchi^1,4^ & Leonard Guarente^2,4^**

1. **Department of Diabetes & Metabolic Diseases, Graduate School of Medicine, The University of Tokyo, Tokyo, Japan**
2. **Department of Biology, Glenn Labs for the Science of Aging, and Koch Institute, MIT, Cambridge MA 02139**
3. **Chromadex, Irvine CA 92618**
4. **Corresponding authors**

**Contact: leng@mit.edu**

**tyamau-tky@umin.net**

**Figure S1**

**Aging reduces ISC number in vivo**

(a)The number of intestinal epithelial cells in villus was quantified in HE staining images from young or old mice (5 mice per group, 25 crypt/villus units per mouse). The representative images are shown in Figure 1a. (b) The representative images of alcian blue staining in the intestine from young or old mice. The quantification of the number of alcian blue positive goblet cells per villus is shown. (6-7 mice per group, approximately 50 crypt/villus units per mouse) (c) The representative images of Chromogranin A staining in the intestine from young or old mice. The quantification of Chromogranin A positive endocrine cells per villus is shown (6 mice per group, approximately 50 crypt/villus units per mouse). (d) The quantification of total BrdU positive cells per crypt as assessed 2 hours after the injection of BrdU into young and old mice (3 mice per group, 50 intact well-orientated crypts units per mouse). The representative images are shown in Figure 1b. (e) Percentage of the LgR5 GFP high cells from the crypts of young and old mice by FACS analysis. The Arrow shows the threshold of GFP intensity for GFP high cells. The same threshold was set up in all samples. (n=11) Original magnifications: X200 (b and c). Scale bar: 50µm (b and c) Values represent the mean ± SEM. *P<0.05; **P<0.01; ***P<0.001; t test.

**Figure S2**

**Aging reduces the formation of intestinal organoids from ISCs ex vivo**

(a) ISCs were isolated from young and old Lgr5-EGFP-IRES-CreERT2 mice (>95% pure) and 2 x 10^3^ cells were cultured in the absence of Paneth cells in culture medium containing 10 µM CHIR. The diameter of more than 30 colonies per each group was analyzed. The representative images are shown in Figure 2b. (b)ISCs and Paneth cells were isolated from young and old Lgr5-EGFP-IRES-CreERT2 mice by flow cytometry and 2 x 10^3^ cells each were co-cultured in the absence of CHIR. The number of colonies was assessed at day 5 (3-4 wells/group). (c) Immunoblotting of SIRT1 or glyceraldehyde phosphate dehydrogenase (GAPDH) in whole intestine lysates or crypt lysates isolated from young and old mice.(d) Immunoblotting of SIRT1 or GAPDH in ISCs isolated from young mice or old mice administered with vehicle or NR (500mg/kg) in drinking water for 6 weeks. Values represent the mean ± SEM. *P<0.05; **P<0.01; ***P<0.001; t test.

**Figure S3**

**RNA seq data from young and old ISCs.**

(A)Regression analysis of average FPKM from RNAseq data obtained from young and old Lgr5 positive cells. Pearson product-moment correlation coefficient r=0.868. (B) Identity of 111 statistically significant differentially expressed genes from RNAseq data.

**Figure S4**

**Nicotinamide riboside (NR) restores the colony formation in aged mice**

(a) Isolated young or old crypts were cultured in medium with or without 1 mM NR as indicated. The bud number from more than 100 organoids per each group was analyzed. Original magnifications: X100. Scale bar: 100µm. (b) Isolated young crypts were cultured in medium with or without 1 mM NR and 1mM rapamycin as indicated (4 wells/group). (c) Isolated young crypts were cultured in medium with or without 1 mM NR and 1 µM EX527 as indicated (3 wells/group). (d) Isolated young ISCs were cultured in medium with or without 1 mM NR and 1mM rapamycin as indicated (3 wells/group). (e) Isolated young ISCs were cultured in medium with or without 1 mM NR and 1 µM EX527 as indicated (3wells/group). Values represent the mean ± SEM. *P<0.05; **P<0.01; ***P<0.001; t test.

**Figure S5**

**In vivo NR treatment restores the organoid formation from ISCs in aged mice**

(a) Crypts from young or old mice administered with vehicle or NR (500mg/kg) in drinking water for 6 weeks were cultured in matrigel. The number of formed organoid colonies was assessed at day 5 (4 wells/group). (b,c) ISCs from young or old mice administered with vehicle or NR were cultured. (b)The number (3 wells/group) and the diameter of colonies (more than 40 colonies/group) were assessed at day 5. (c) The number (3 wells/group) and the bud number of formed organoids (not spheres) (more than 60 colonies/group) were assessed at day 9. (d) Paneth cells were isolated from young or old mice administered with vehicle or NR were co-cultured with ISCs from young mice in the medium containing 10 µM CHIR. The number (4 wells/group) and the diameter of colonies (more than 40 colonies/group) were assessed at day 5. Y:Young, O:Old. Original magnifications: X50 (a,b,c and d),Scale bar: 100µm (a, b and d); 200µm (c)

**Figure S6**

**NR treatment restores ISC number in aged mice *in vivo***

(a) H & E staining images and the quantification of villi sizes in the intestine of young or old mice administered with vehicle or NR (8 mice per group, approximately 100 crypt/villus units per mouse). (b) In situ pS6 staining images in the intestine of old mice administered with vehicle or NR (3 mice per group). (arrowheads: ISCs adjacent to Paneth cells) Original magnifications: X100 (a); X200 (b). Scale bar: 50µm (a and b).Values represent the mean ± SEM. *P<0.05; **P<0.01; ***P<0.001; t test.
